# The Difference between Right and Wrong: Accuracy of Older and Younger Adults’ Story Recall

**DOI:** 10.3390/ijerph120910861

**Published:** 2015-09-02

**Authors:** Danielle K. Davis, Nicole Alea, Susan Bluck

**Affiliations:** 1 Department of Psychology, University of Florida, P.O. Box 112250, Gainesville, FL, 32611-225, USA; E-Mail: dstahldavis@ufl.edu; 2 Psychology Unit, Department of Behavioural Sciences, Faculty of Social Sciences, University of the West Indies, St. Augustine, Trinidad and Tobago

**Keywords:** fictional text recall, storytelling, accuracy, memory

## Abstract

Sharing stories is an important social activity in everyday life. This study used fine-grained content analysis to investigate the accuracy of recall of two central story elements: the gist and detail of socially-relevant stories. Younger (*M* age = 28.06) and older (*M* age = 75.03) American men and women (*N* = 63) recalled fictional stories that were coded for (i) accuracy of overall gist and specific gist categories and (ii) accuracy of overall detail and specific detail categories. Findings showed no age group differences in accuracy of overall gist or detail, but differences emerged for specific categories. Older adults more accurately recalled the gist of *when* the event occurred whereas younger adults more accurately recalled the gist of *why* the event occurred. These differences were related to episodic memory ability and education. For accuracy in recalling details, there were some age differences, but gender differences were more robust. Overall, women remembered details of these social stories more accurately than men, particularly *time* and *perceptual* details. Women were also more likely to accurately remember the gist of *when* the event occurred. The discussion focuses on how accurate recall of socially-relevant stories is not clearly age-dependent but is related to person characteristics such as gender and episodic memory ability/education.

## 1. Introduction

Sharing stories is an everyday memory activity common to most humans [1,2]. Of particular interest in the study of aging is how the types of stories that people tell, and how they tell them, changes across the lifespan. Differences in storytelling ability and style have been related to psychosocial and biological changes that accompany aging [3,4]. Stories told in everyday life may be about autobiographical events [5,6,7,8]. Individuals also, however, regularly share non-autobiographical stories such as retelling interesting stories they have read (e.g., prose recall) [9,10], sharing stories about other people (e.g., gossip) [11,12], or telling friends and co-workers stories they have heard reported in the media (e.g., the news) [13]. Prior research investigating story recall has revealed differences in the amount of information that younger and older adults express [14,15] as well as the amount of accurate information produced [16]. The primary goal of the present study was to examine whether age is related to how *accurately* individuals remember the *gist* and *detail* in stories (*i.e.*, non-autobiographical). We also examined the role of other personal characteristics, gender and episodic memory ability/education, on recall accuracy.

Moving beyond past research, the present study examined accuracy quite comprehensively. Overall accuracy of recalled information was assessed but we also decomposed gist and detail into distinct categories (gist: *who*, *what*, *where*, *when*, and *why*; details: *place*, *time*, *perceptual information*, and *emotion/thought information*). Below we discuss the importance of accuracy in story recall and then review age and gender differences in recall, drawing from literatures that have examined accuracy.

### 1.1. The Role of Accuracy in Story Recall

How important is it to accurately recall events we hear about or read about in everyday life (*i.e.*, non-autobiographical events)? Accuracy can sometimes be quite consequential, such as when individuals’ autobiographical recall involves providing eyewitness testimony in a courtroom [17,18,19] or choosing a perpetrator from a police line-up [20,21]. Eyewitness testimony and line-up identification, however, are stressful events that are not common parts of individuals’ everyday lives [22].

In many situations, the accuracy of our recall is not so clearly consequential. Being at least mostly accurate is usually important but recalling with complete accuracy all of the specific details of an event, whether autobiographical or non-autobiographical, is not always required in daily life. For example, in some situations, a conversation regarding an event that happened in the world may be geared toward entertaining the current listener. At other times, telling of the story focuses on the symbolic, deeper meaning, take-home moral of the event, or how it coheres with the larger story [14,23]. In such cases, accuracy of *literal* details of the event may be less of a priority.

In some everyday situations, however, conversation may indeed be focused on accurate recall, such as when one individual tells a story about another person to a third party. When accurate, such story-sharing can be prosocial, strengthening social bonds and allowing group members to be informed about one another’s lives [12,24]. When the information is unreliable or false, however, it may have significant social consequences including the violation of interpersonal trust, weakening of relationships, or damages to a person’s social reputation [25].

In sum, research suggests that the importance of being accurate in recalling events that we hear or read about is dictated, at least partly, by social context. The balance between accuracy and flexibility is what makes the human memory system so sophisticated [26,27,28]. Storytelling is inherently social and its ultimate social function may be driven by person-environment fit in a specific life circumstance [29]. The type of story (*i.e.*, autobiographical *versus* non-autobiographical) and the purpose of telling the story (*i.e.*, to entertain *versus* to provide testimony) may influence the level of necessary accuracy. There are also, however, person-specific characteristics such as age and gender that may partially govern the amount of accurate information produced when people recall and retell events.

### 1.2. Age Differences in Story Recall

Several studies have examined age differences in story recall. The most common method is to assess traditional elements of stories: *gist* and *detail*. Gist involves the central features of an event, including remembering *who*, *what*, *where*, *when*, and *why* [30]. Individuals often expand on the basic gist by including *details* [7,31,32,33,34]. Most research on age differences has focused on how much individuals can recall about an event. Such research suggests that older adults tend to recall more of the gist [29,35,36] while younger adults recall more specific details [7,37,38,39,40,41,42,43]. Specifically, older adults recall fewer details related to where the event took place, as well as perceptions, thoughts, and emotions of individuals in the story [7]. Note, however, that the *accuracy* of the recalled gist and detail is not typically the focus of these studies. In the few instances in which accuracy of story recall has been examined, older adults recall gist better than detail and are overall less accurate than younger adults when recalling surface level details [16,23].

Though age differences in accuracy have not received much attention generally, they have been a major focus in the false memory and eyewitness memory literatures. For example, some studies suggest that older adults are more likely than younger adults to falsely recall the source of a memory or develop a false memory ([44,45,46]; but see [47,48]). Similarly, in identifying faces from a police line-up, false alarm rates (*i.e.*, recognizing new faces as already seen) is higher in older compared to younger adults [49,50,51,52]. Older adults’ less accurate recall has been attributed to cognitive abilities such as poorer source monitoring ability [46] and declines in episodic memory [53]. In the current study we investigated whether these aging effects for accuracy in recall would extend from eyewitness reporting situations to socially-relevant stories as might be heard and told in daily life. We also explore whether age differences in cognitive status (*i.e.*, episodic memory) as well as education level might explain any obtained age differences.

### 1.3. Gender Differences in Story Recall

Men and women age differently; they are subject to different psychosocial factors throughout the lifespan [54]. Regardless of age, however, people adopt different social roles based on gender [55,56,57] that may in turn influence how they recall stories—particularly stories about social events—as well as the accuracy of those recollections. Past research provides support for this idea. For example, women have more accurate prose recall memory [58] and husbands often rely on their wives for cues when recalling shared events [59]. Woman may also make better eyewitnesses. For example, when Areh [60] showed men and women short recordings of a robbery, women had more accurate memories for the event than men overall, with a specific advantage for information related to a social component, the appearance of the assailant and the victim. This research is consistent with other studies showing women are more accurate eyewitnesses, particularly for person descriptions and characteristics [61,62,63,64]. Likewise, women are more accurate at face recognition than men [65].

One explanation for gender differences in accuracy when recalling story information might be that women adopt a social role in which they feel responsible for remembering and disseminating interpersonal information. Thus, accuracy may be more important to women than men during story recall. Women’s advantage for accurately recalling stories may also, however, be related to their having better episodic memory as compared to men, specifically verbal memory [66,67,68]. Women have also been theorized to have more elaborate categories or engage in more comprehensive processing for person information [60,61,69]. Thus, it seems that one’s gender, not only their age, may affect the importance placed on recalling story information accurately. As such, an exploratory aim of the present study was to examine gender differences in accuracy of story recall.

### 1.4. The Present Study

The goal of the present study was to examine age group differences in storytelling of socially-relevant events. Specifically, we examined recall of non-autobiographical stories commonly shared in everyday life (*i.e.*, prose, gossip, news). Such stories can be readily compared against a correct version of the original experimenter-provided text to ascertain accuracy. Younger and older adults heard stories and were asked to recall and narrate them. Narratives were then content-coded. We took a nuanced approach by coding for the overall accuracy of recall of the gist and detail of events but also precisely assessing components of these story elements. Doing so allowed for a fine-grained examination of age group differences. That is, we examined if age differences in accurate recall were specific to particular types of information in the story (e.g., recalling the gist of *who* was in the event, or recalling details related particularly to characters’ *emotions and thoughts*).

The two specific aims of the study were to investigate age differences in overall accuracy of recall of gist information (1a) and accuracy of specific categories of gist (1b), as well as age group differences in the overall recall of detail information (2a) and specific categories of detail (2b). For all specific aims, gender was included in analyses so as to explore this additional person characteristic and how it might affect accuracy of recall. Additionally, as cognitive status is frequently given as a reason for age [46,53] and gender [66] differences in the accuracy of recall, we also examined whether any obtained differences held when variables that have been shown to relate to cognitive functioning—specifically, episodic memory and education—were used as covariates in analyses [70].

## 2. Method Section

### 2.1. Participants

Thirty-two younger (16 males; *M* = 28.06 years; *SD* = 5.24) and 31 older (16 males; *M* = 75.03 years; *SD* = 6.22) adults participated in the study. Participant ethnicity reflected the make-up of the community in which the study was completed [71]: 72% of younger adults were Caucasian, 13% Hispanic, 6% Asian, 6% Black, and 3% reported their ethnicity as ‘other’. Ninety-four percent of older adults reported their ethnicity as Caucasian. No ethnicity differences were detectable on study measures. Younger adults were recruited from the campus or surrounding community and older adults were recruited from community organizations and screened for cognitive impairment [72]. On a Likert-scale [73] comparing one’s own health to same-aged peers, with responses ranging from 1 (*very good*) to 6 (*very poor*), there were no age (young *M* = 1.81, *SD* = 0.86; old *M* = 1.94, *SD* = 1.03), *t*(61) = 0.27, *p* = 0.61, or gender differences, (men *M* = 2.06, *SD* = 0.91; women *M* = 1.67, *SD* = 0.95), *t*(61) = 2.71, *p* = 0.10.

### 2.2. Measures and Procedure

Participants completed a background questionnaire to measure years of education, as well as an episodic memory ability measure, which is thought to be related to story recall [74]. Episodic memory ability was assessed using the Auditory Verbal Learning Task (AVLT) [75]. In this task, participants listened to 15 semantically unrelated words and were then asked to immediately write down as many of the words as they could remember. Only one trial of the AVLT was used. The ALVT was scored as the number of words correctly remembered.

Participants were then asked to remember two socially-relevant, non-autobiographical fictional stories: a story about a couple having a romantic evening and a story about a couple taking a vacation. These fictional stories were developed for and have been commonly used in research with younger and older adults [1,59]. The events are likely to have been experienced by both younger and older men and women and are written in a colloquial style. They describe a single event, include information about the characters’ intentions, plans, evaluations, outcomes, and behavior, and are reported as being moderately emotional stories that elicit positive feelings and are somewhat interesting and true-to-life by both younger and older adults [76]. The order of recalling events was counterbalanced across age and gender, and there were no order effects.

The story-recall portion of the procedure began by presenting participants with a three-minute pre-recorded narrative about one of the events (romantic evening or vacation). After the story was presented, participants were given 10 min to orally narrate the fictional story. This procedure was then repeated for the other event. Participants recalled the story to a young female interviewer to enhance disclosure [77]. The interviewer was trained to act as an interested listener, responding with engaging facial expressions and small agreements (“umm hmm”, oh, *etc.*), but was trained to not interact verbally with participants beyond the use of three standard probes that were intended to elicit further recall once the participant appeared to be finished recalling their story. These probes consisted of: “Can you tell me more about what they were doing, thinking or feeling?”, “Is there anything else?”, and “Is that all?”. Participants’ oral narration was audio-recorded.

### 2.3. Content-Coding for Accuracy

Verbatim text transcripts of participants’ audio-recorded memory stories were created. Transcripts were cleaned for extraneous speech fillers (e.g., um, uh) prior to analyzing the accuracy of story recall. Coders were trained using pilot data. Reliability between two coders was achieved for each code using a sub-sample of 15% of the narratives. Discrepancies and coder drift [78] were addressed in weekly meetings. Coders did not have any knowledge of the study aims, nor the age or gender of the participants. All narratives were coded to identify the accuracy of the gist and details that were explicitly expressed in participants’ recall of the stories. Each coding scheme is briefly described below (manuals available upon request). Table 1 summarizes the codes and provides examples from participants’ recalled stories.

**Table 1:** Examples of coding schemes for accurate information recalled for gist and detail.

Code	Accurate Original Story Information
*Gist*	
What	browsed around at the booths parked the car away from the event
Who	the couple
Where	in Washington, DC stopped in Kansas along the way
When	during the evening in Fall
Why	because she had wanted to go because it was a holiday
*Details*	
Place	area around the Mall near the ranger station
Time	it was the 4th of July in August
Perceptual	fireworks were spectacular drive was cool and comfortable
Emotion/Thought	impressed with the evening happy about their first trip

#### 2.3.1. Accuracy of Gist

The content-coding scheme for accuracy of gist was developed by the authors based on a comprehensive list of accurate gist-like responses that appear in the original manual from which the stories were drawn [76]. Five gist categories were identified, including: (i) *what* happened; (ii) *who* the characters were; (iii) *where* the story took place; (iv) *when* the event occurred; and (v) *why* the events in the fictional story happened (e.g., motivations, intentions, and attributions concerning why the event occurred). All of these canonical features appeared in the stories that participants heard. Inter-rater agreement for the five categories was: *what* = 100%, *who* = 90%, *where* = 95%, *when* = 85%, and *why* = 90%. Kappa ranged from 0.71 to 1.00.

Coding occurred at the global level. That is, each narrative was read in its entirety to determine whether or not the coded gist was accurate. A category was considered accurate (a score of 1) if the participants’ story contained phrases that correctly gave the gist (*i.e.*, matched the original story for that category) and inaccurate (a score of 0) if the recalled narrative inaccurately represented the gist of that category. The dependent variable for total gist was the number of gist categories correctly recalled across the two fictional stories. The total possible gist accuracy score across both narratives ranged from 0 (no accurate gist for any category) to 10 (accurate gist for all categories across both stories). The gist score for each individual gist category ranged from 0 to 2 out of a possible 2 (*i.e.*, gist category accurate in both stories).

#### 2.3.2. Accuracy of Detail

The content-coding scheme for accuracy of detail was developed by the authors but relied heavily on detail categories used in previous literature [7], including: (i) *place* details, which referred to information about where the event occurred; (ii) *time* details, which referred to specifics about when the event occurred; (iii) *perceptual* information, which referred to details about the auditory and visual landscape of the event; and (iv) *emotion/thought* details, including specific information about the feelings and internal states of the characters in the story. To begin, a checklist of all details in each of the categories that appeared in the original fictional stories was developed. Participants recalled stories were then compared to this checklist by independent coders. Each detail was thereby coded as either accurate or as an error. Three types of errors were possible: errors of omission (*i.e.*, a detail appeared in the original story but was not included in the participant’s story), change errors (*i.e.*, details from the original story were recalled in the participant’s story but were recalled inaccurately), and errors of commission. Commission involves new details that the participant recalled but that were not actually part of the original story they heard (*i.e.*, the detail was fabricated). This type of error occurred with such low frequency (0.01%) that it was not analyzed. Further, change errors also occurred with very low frequency (12%). Although analyzed, no significant results were found, perhaps due to a floor effect. As a result, the accuracy data reported in analyses are errors of omission only. Inter-rater agreement for identifying whether a detail was accurate or an error within each detail category were: *place* = 80%, *time* = 86%, *perceptual* = 90%, and *emotion/thought* = 81%. Kappa ranged from 0.68 to 0.81. The accuracy of (i) recalled details and (ii) omission errors made are reported as a proportion out of the total number of details possible based on the check list. As would be expected, these two coding categories were highly negatively related, *r* (60) = −0.93, *p* < 0.001, but not completely overlapping.

## 3. Results and Discussion

### 3.1. Preliminary Analyses

Preliminary analyses were conducted to examine the relation between possible covariates, including episodic memory ability and education, with age, gender, overall accuracy of gist, and accuracy of and error in the details recalled from the fictional memory stories. Age was kept as a grouped variable (younger/older) to correspond with other analyses, so both gender (1 = male, 2 = female) and age (1 = young, 2 = old) variables were dummy coded. A point bi-serial correlation was used for the categorical variables, and Pearson’s correlation used for all others. The resultant correlation matrix appears in Table 2. Variables were considered as covariates if related to (i) either age or gender or (ii) overall accuracy of gist, overall accuracy of details, or overall proportion of errors made. As can be seen in Table 2, older adults had poorer episodic memory ability than younger adults and women had better episodic memory ability than men. Although episodic memory ability was not related to overall accuracy of recalled gist, it was positively related to the overall accuracy of details recalled and negatively related to overall proportion of errors made. Having better episodic memory ability was related to more accurately recalling the details of stories and making fewer errors. Older adults reported fewer years of education than younger adults. Education, however, was not related to the overall accuracy of gist or the proportion of accurate or inaccurate details. Analyses were thus conducted both with and without episodic memory and education as covariates [79].

**Table 2:** Correlations between person characteristics, education, episodic memory and recall accuracy.

	Measure	1	2	3	4	5	6	7
1	Age Group	-						
2	Gender	−0.02	-					
3	Education in total years	−0.36 **	0.08	-				
4	Episodic Memory	−0.32 *	0.37 **	0.20	-			
5	Gist Accuracy Total	−0.11	0.21	0.19	0.14	-		
6	Detail Accuracy Total	−0.24	0.34 **	0.15	0.34 **	0.20	-	
7	Detail Missing Total	0.28 *	−0.38 **	−0.23	−0.34 **	−0.22	−0.93 **	-

* *p* < 0.05, ** *p* < 0.01

### 3.2. Primary Analyses

All analyses were first conducted without covariates in the model, and then reanalyzed with episodic memory ability and education as covariates. Descriptive statistics for cell means without covariates in the model for accuracy of gist, details accurately remembered from the stories, and errors of omission for details (both overall and for the categories, in all cases) are reported in Table 3, Table 4 and Table 5, respectively. Figures highlight statistically significant effects. Estimated marginal means and standard errors are reported when covariates were included in the model. Full statistics are not reported for the models with covariates if non-significant results remained unchanged. The covariates in the models are evaluated at: episodic memory *M* = 8.26; education *M* = 17.51 years.

#### 3.2.1. Gist Accuracy

The accuracy of gist recalled from the stories overall and gist recalled in each category by age and gender are reported in Table 3. A 2 (age) x 2 (gender) analysis of variance (ANOVA) was conducted on the overall accuracy of gist. With no covariates included, as can be seen, there were no age, *F* < 1.00, or gender main effects, *F* (1, 58) = 2.74, *MS* = 3.39, *p* = 0.10, *ηρ²* = 0.04, nor an interaction, *F* < 1.00, for the overall accuracy of gist remembered from the stories. These non-significant effects remained when episodic memory ability and education were included as covariates in the model.

**Figure 1:**
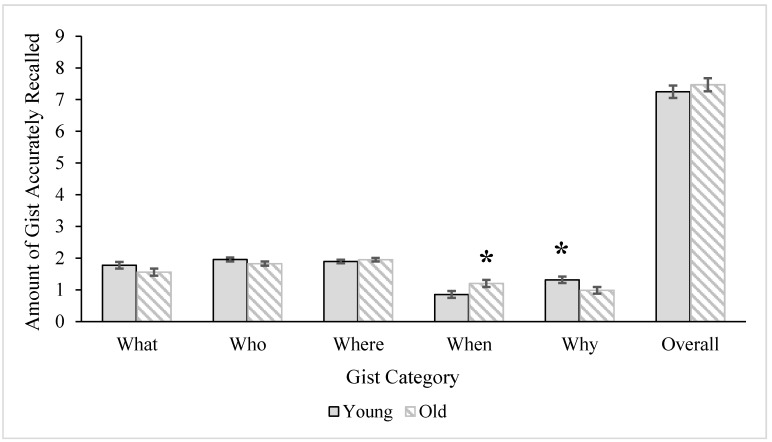
Age group differences for overall gist accuracy and accuracy of gist categories. Error bars represent ± 1SE. Asterisks mark significant effects at the *p* < 0.05 level. Age differences are no longer significant after including covariates (episodic memory, education) in the model.

**Figure 2:**
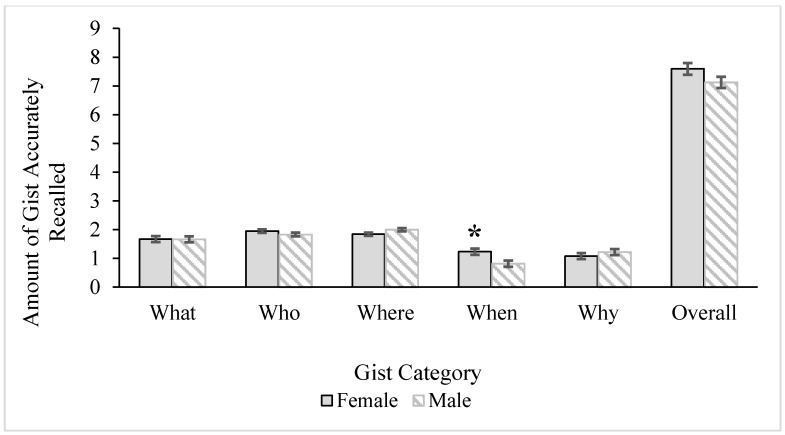
Gender differences for overall accuracy of gist and gist categories. Error bars represent ± 1SE. Asterisk marks significant effect at the *p* < 0.01 level. Gender differences remained significant after including covariates (episodic memory, education) in the model.

However, a 2 (age) x 2 (gender) multivariate analysis of variance (MANOVA) conducted to examine the accuracy within each category of gist (*what*, *who*, *where*, *when*, and *why* the events in the stories that participants heard had occurred) revealed significant effects. The multivariate age group, Wilks’ *Λ* = 0.75, *F* (5, 39) = 2.60, *p* = 0.04, *ηρ²* = 0.25, and gender, Wilks’ *Λ* = 0.68, *F* (5, 39) = 3.61, *p* = 0.009, *ηρ²* = 0.32, main effects were significant. The multivariate interaction effect was not, Wilks’ *Λ* = 0.85, *F* (5, 39) = 1.33, *p* = 0.27, *ηρ²* = 0.15. Descriptive statistics for the accuracy of the gist categories, as well as overall gist, by age and gender are illustrated in Figure 1 and Figure 2, respectively. There was a significant age group difference for the accuracy of recalling *when* the event occurred, *F* (1, 43) = 5.02, *MS* = 0.39, *p* = 0.03, ηρ² = 0.11, and *why* the event occurred, *F* (1, 43) = 5.13, *MS* = 1.27, *p* = 0.03, *ηρ²* = 0.11. As can be seen in Figure 1, older adults more accurately recalled *when* the event occurred compared to younger adults, whereas younger adults more accurately remembered *why* the event occurred. There were no age group differences for the accuracy of the *what*, *F* (1, 43) = 2.11, *MS* = 0.55, *p* = 0.15, *ηρ²* = 0.05, *who*, *F* (1, 43) = 2.06, *MS* = 0.19, *p* = 0.16, *ηρ²* = 0.05, or *where*, *F* < 1.00, categories of gist. There was one significant univariate effect for gender for the accuracy of recalling *when* the event occurred, *F* (1, 43) = 7.30, *MS* = 2.02, *p* = 0.01, *ηρ²* = 0.15. As seen in Figure 2, women more accurately remembered when the event occurred compared to men. No other gender differences were significant, *what*: *F* < 1.00; *who*: *F* (1, 43) = 1.78, *MS* = 0.17, *p* = 0.19, *ηρ²* = 0.04, *where*: *F* (1, 43) = 3.77, *MS* = 0.29, *p* = 0.06, *ηρ²* = 0.08, *why*: *F* < 1.00.

**Table 3:** Overall and category accuracy of gist recall by age and gender.

Gist Accuracy	Age Group							
	Younger Adults				Older Adults			
	Women		Men		Women		Men	
	*M*	*SD*	*M*	*SD*	*M*	*SD*	*M*	*SD*
Overall	7.69	1.19	7.25	1.29	7.50	0.94	7.00	0.97
What	1.64	0.63	1.90	0.30	1.70	0.48	1.42	0.51
Who	2.00	0.00	1.91	0.30	1.90	0.32	1.75	0.45
Where	1.79	0.43	2.00	0.00	1.90	0.32	2.00	0.00
When	1.07	0.47	0.64	0.50	1.40	0.52	1.00	0.60
Why	1.36	0.49	1.27	0.47	0.80	0.42	1.17	0.58

*Note*: Descriptive statistics are without covariates in the model. The total possible gist accuracy score across both narratives ranged from 0 (no accurate gist for any category) to 10 (accurate gist for all categories across both stories). The gist score for each individual gist category ranged from 0 to 2 out of a possible 2.

The MANOVA was rerun including covariates (episodic memory, education) in the model. The multivariate age group effect went away, Wilks’ *Λ* = 0.89, *F* < 1.00, but the gender multivariate main effect remained significant, Wilks’ *Λ* = 0.65, *F* (5, 37) = 3.92, *p* = 0.006, *ηρ²* = 0.35. Again, the univariate effect for gender emerged for accurately remembering the gist for *when* the event occurred, *F* (1, 41) = 10.81, *MS* = 2.78, *p* = 0.002, *ηρ²* = 0.21. Women (*M* = 1.28, *SE* = 0.11) were more likely to accurately remember the gist of *when* the event occurred compared to men (*M* = 0.77, *SE* = 0.11), even when controlling for women’s better episodic memory and higher education.

In sum, although there were no age or gender differences in overall accuracy of gist in story recall, there were differences by gist category. Younger adults more accurately remembered *why* the event in the story occurred, whereas older adults more accurately remembered *when* the event in the story occurred. However, these differences were eliminated when controlling for episodic memory ability and education level. The gender difference for accurately remembering *when* the event occurred remained regardless of covariates: Women remember more accurately *when* the event occurred compared to men.

#### 3.2.2. Details: Accuracy

**Figure 3:**
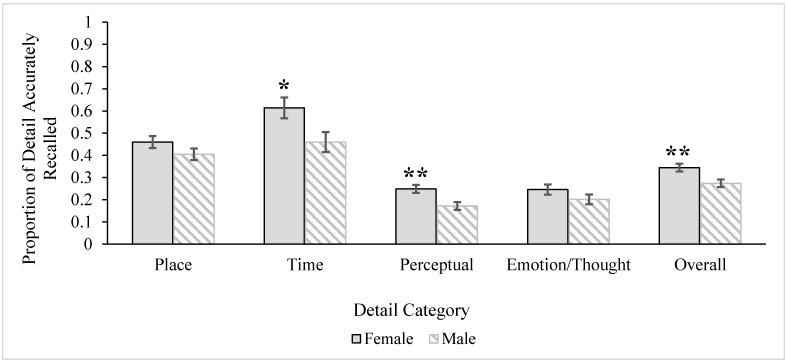
Gender differences for accuracy of overall detail and detail categories. Error bars represent ± 1SE. Single asterisk marks significant effect at the *p* < 0.05 level and double asterisks mark significant effects at the *p* < 0.01 level. Gender differences for overall gist remained significant after including covariates (episodic memory, education) in the model, but multivariate effects (*time* and *perceptual*) became N.S.

A 2 (age) x 2 (gender) ANOVA was conducted to examine age and gender differences in the overall accuracy of details recalled from stories. Descriptive statistics by age and gender for the accuracy of details recalled overall and by detail category are reported in Table 4. There was no significant main effect for age, *F* (1, 58) = 3.46, *MS* = 0.03, *p* = 0.07, *ηρ²* = 0.06, but there was a significant gender main effect, *F* (1, 58) = 7.81, *MS* = 0.08, *p* = 0.007, *ηρ²* = 0.12. As seen in Figure 3, women recalled a higher proportion of accurate details overall compared to men. The age x gender interaction was not significant, *F* < 1.00. The gender difference in the accuracy of overall detail remained significant when controlling for education and episodic memory, *F* (1, 56) = 3.99, *MS* = 0.04, *p* = 0.05, *ηρ²* = 0.07 (women *M* = 0.34, *SE* = 0.02; men *M* = 0.28, *SE* = 0.02). Non-significant age and interaction effects remained non-significant with covariates in the models.

**Table 4:** Overall and category accuracy of detail recall by age and gender.

Detail Accuracy	Age Group							
	Younger Adults				Older Adults			
	Women		Men		Women		Men	
	*M*	*SD*	*M*	*SD*	*M*	*SD*	*M*	*SD*
Overall	0.36	0.09	0.30	0.12	0.32	0.06	0.25	0.10
Place	0.45	0.13	0.42	0.19	0.47	0.09	0.39	0.15
Time	0.63	0.21	0.51	0.27	0.60	0.23	0.41	0.29
Perceptual	0.28	0.19	0.20	0.11	0.22	0.08	0.14	0.08
Emotion/Thought	0.29	0.14	0.23	0.14	0.19	0.07	0.18	0.12

*Note*: Descriptive statistics are without covariates in the model. Means and SEs above represent proportions of accurately recalled details out of the total possible number of to-be-recalled details.

A 2 (age) x 2 (gender) MANOVA was conducted with the accuracy of the categories of detail (*i.e.*, *place*, *time*, *perceptual information*, *emotion/thought* details) as the dependent variables. The multivariate age main effect was not significant, Wilks’ *Λ* = 0.86, *F* (4, 55) = 2.26, *p* = 0.07, *ηρ²* = 0.14, but the multivariate gender effect was, Wilks’ *Λ* = 0.83, *F* (4, 55) = 2.81, *p* = 0.03, *ηρ²* = 0.17. The multivariate interaction was not significant, *F* < 1.00. Univariate tests revealed significant gender differences in the accuracy of recalling *time*, *F* (1, 58) = 5.64, *MS* = 0.37, *p* = 0.02, *ηρ²* = 0.09, and *perceptual* details, *F* (1, 58) = 9.26, *MS* = 0.09, *p* = 0.004, *ηρ²* = 0.14, which are illustrated in Figure 3. As can be seen, women more accurately recalled both *time* and *perceptual* details than men. The gender difference for the accuracy of *place*, *F* (1, 58) = 2.14, *MS* = 0.05, *p* = 0.15, *ηρ²* = 0.04, and *emotion/thought* details, *F* (1, 58) = 1.92, *MS* = 0.03, *p* = 0.17, *ηρ²* = 0.03, were not significant.

The multivariate gender difference in the accuracy of the detail categories was not maintained when education and episodic memory covariates were included in the model, Wilks’ *Λ* = 0.89, *F* (4, 53) = 1.67, *p* = 0.17, *ηρ²* = 0.11. Interestingly, however, when education and episodic memory covariates were included, a multivariate age main effect emerged, Wilks’ *Λ* = 0.81, *F* (4, 53) = 3.13, *p* = 0.02, *ηρ²* = 0.19. There was a univariate age effect for the accuracy of *emotion/thought* details, *F* (1, 56) = 5.35, *MS* = 0.08, *p* = 0.02, *ηρ²* = 0.09. Younger adults (*M* = 0.27, *SE* = 0.02) accurately remembered a higher proportion of *emotion/thought* details compared to older adults (*M* = 0.18, *SE* = 0.02) when episodic memory ability and education levels were controlled. There were no age group differences for the accuracy of *place* or *time* details, *F*s < 1.00, or for *perceptual* details, *F* (1, 56) = 3.85, *MS* = 0.04, *p* = 0.06, *ηρ²* = 0.06.

To summarize, analyses revealed both age and gender differences in the accuracy of details recalled. Women overall recalled a higher proportion of accurate details compared to men. Multivariate analyses revealed that this difference was driven by two specific categories: *time* and *perceptual* details. However, gender effects disappeared when education and episodic memory covariates were included in the model. Alternatively, age effects emerged when education and episodic memory covariates were included in the model: Younger adults remembered more *emotion/thought* details than older adults.

#### 3.2.3. Details: Errors of Omission

**Figure 4:**
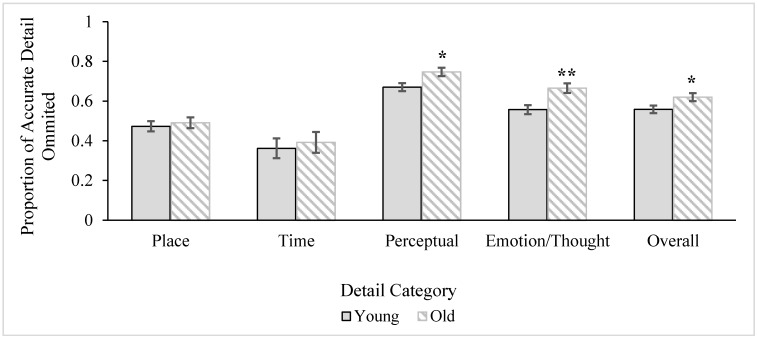
Age differences in errors of omission for details overall and by category. Bars represent ± 1SE. Single asterisks mark significant effects at the *p* < 0.05 level and double asterisk marks significant effect at the *p* < 0.01 level. Age differences in overall detail disappeared after including covariates (episodic memory, education) in the model, but multivariate effects (*perceptual* and *emotion/thought*) remained significant.

**Figure 5:**
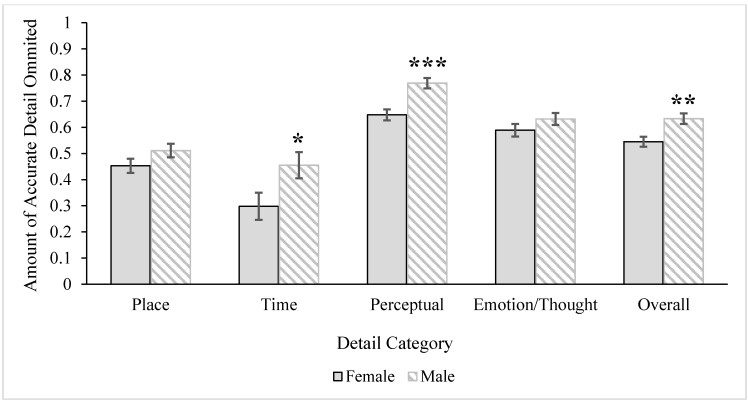
Gender differences in errors of detail omission overall and by category. Bars represent ± 1SE. Single asterisk marks significant effect at the *p* < 0.05 level, double asterisk marks significant effect at the *p* < 0.01 level, and triple asterisk marks significant effect at the *p* < 0.001 level. All effects remain significant except for the *time* gender difference after including covariates (episodic memory, education) in the univariate and multivariate models.

In addition to the number of errors overall, we also examined age differences in the frequency of omission errors in order to mirror what is typically done in the episodic memory literature [80,81,82]. Again, a 2 (age) x 2 (gender) ANOVA, followed by a MANOVA, for the overall proportion of errors made and errors made for each category of details (respectively) were conducted, followed by the same analyses rerun with covariates included. Descriptive statistics for this analysis are presented in Table 5. For the overall proportion of errors made when recalling details, there was a main effect of age, *F* (1, 58) = 5.08, *MS* = 0.06, *p* = 0.03, *ηρ²* = 0.08, and a main effect of gender, *F* (1, 58) = 10.21, *MS* = 0.12, *p* = 0.002, *ηρ²* = 0.15. As seen in Figure 4, older adults had a higher proportion of errors of omission for overall detail compared to younger adults. Men omitted more details overall compared to women, illustrated in Figure 5. There was no interaction, *F* < 1.00. However, when education and episodic memory covariates were included in the model, the age group differences disappeared, *F* (1, 56) = 1.95, *MS* = 0.02, *p* = 0.17, *ηρ²* = 0.03. The gender difference remained significant, *F* (1, 56) = 5.71, *MS* = 0.07, *p* = 0.02, *ηρ²* = 0.09: Again, men (*M* = 0.63, *SE* = 0.02) had a higher proportion of errors of omission than women (*M* = 0.55, *SE* = 0.02) for overall details, even when controlling for gender differences in episodic memory ability and education.

The MANOVA examining errors of omission for the categories of detail revealed significant age, Wilks’ *Λ* = 0.77, *F* (4, 55) = 4.10, *p* = 0.006, *ηρ²* = 0.23, and gender, Wilks’ *Λ* = 0.76, *F* (4, 55) = 4.45, *p* = 0.003, *ηρ²* = 0.24, multivariate main effects, but no interaction, Wilks’ *Λ* = 0.91, *F* (4, 55) = 1.29, *p* = 0.29, *ηρ²* = 0.08. The univariate tests revealed that the age differences were for *perceptual*, *F* (1, 58) = 7.04, *MS* = 0.09, *p* = 0.01, *ηρ²* = 0.11, and *emotion/thought* details, *F* (1, 58) = 10.58, *MS* = 0.18, *p* = 0.002, *ηρ²* = 0.15, but not for *place* or *time* details, *F*s < 1.00. As also seen in Figure 4, older adults omitted a higher proportion of both *perceptual* and *emotion/thought* details compared to younger adults. The univariate tests for gender were significant for both the omission of *time*, *F* (1, 58) = 4.82, *MS* = 0.38, *p* = 0.03, *ηρ²* = 0.08, and *perceptual*, *F* (1, 58) = 17.09, *MS* = 0.22, *p* < 0.001, *ηρ²* = 0.23, details, where men had a higher proportion of omitted time and perceptual details compared to women (see Figure 5). There were no gender differences in the omission of *place*, *F* (1, 58) = 2.33, *MS* = 0.05, *p* = 0.13, *ηρ²* = 0.04, or *emotion/thought* details, *F* (1, 58) = 1.69, *MS* = 0.03, *p* = 0.20, *ηρ²* = 0.03. The multivariate age, Wilks’ *Λ* = 0.74, *F* (4, 53) = 4.75, *p* = 0.002, *ηρ²* = 0.26, and gender, Wilks’ *Λ* = 0.79, *F* (4, 53) = 3.61, *p* = 0.01, *ηρ²* = 0.21) effects remained when education and episodic memory covariates were included in the model. For age, the pattern of results was the same: Older adults omitted a higher proportion of *perceptual*, *F* (1, 56) = 4.76, *MS* = 0.06, *p* = 0.03, *ηρ²* = 0.08, and *emotion/thought*, *F* (1, 56) = 9.01, *MS* = 0.15, *p* = 0.004, *ηρ²* = 0.14, details compared to younger adults. For gender, however, the difference between men and women for the omission of *time* details went away when controlling for education and episodic memory covariates, *F* (1, 56) = 1.04, *MS* = 0.07, *p* = 0.31, *ηρ²* = 0.02, but the difference for *perceptual* details remained, *F* (1, 56) = 13.49, *MS* = 0.18, *p* = 0.001, *ηρ²* = 0.19. Men (*M* = 0.77, *SE* = 0.02) continued to have a higher proportion of omissions for perceptual details compared to women (*M* = 0.63, *SE* = 0.02).

**Table 5:** Descriptive statistics for errors of omission for details overall and by category.

Errors of Omission	Age Group							
	Younger Adults				Older Adults			
	Women		Men		Women		Men	
	*M*	*SD*	*M*	*SD*	*M*	*SD*	*M*	*SD*
Overall	0.52	0.13	0.59	0.12	0.57	0.06	0.67	0.10
Place	0.46	0.17	0.48	0.17	0.44	0.09	0.54	0.15
Time	0.33	0.24	0.39	0.24	0.27	0.24	0.52	0.38
Perceptual	0.61	0.14	0.73	0.15	0.69	0.07	0.81	0.08
Emotion	0.52	0.14	0.59	0.15	0.66	0.08	0.66	0.13

*Note*: Descriptive statistics are without covariates in the model. Means and standard deviations shown represent the proportion of omitted details out of the total possible number of to-be-recalled details.

Thus, results revealed that older adults made more errors of omission overall than younger adults, but multivariate analyses illustrated that this effect occurred specifically for *perceptual* and *emotion/thought* details. These effects remained when including covariates in the model. For effects of gender, men omitted more details overall than women, and this effect remained significant after accounting for education and episodic memory covariates. Additional multivariate analyses revealed that this gender difference specifically occurred for *time* details (without covariates) as well as *perceptual* details (with and without covariates).

### 3.3. Discussion

Individuals of all ages tell stories as part of their daily lives [83]. Stories can sometimes include fictional events pertaining to famous characters (e.g., a TV character’s wedding) or individuals in our own lives (e.g., telling one friend about another friend’s visit to the doctor).  More generally, telling stories can play an important part in fostering social relationships [84]. Past research examining age differences in fictional story recall has generally focused on the *expression* of gist and detail, showing that older adults tend to perform better on gist recall [30,35] whereas younger adults tend to focus on the recall of the story’s details [37,38,42]. The predominant goal in the present study was not just to observe how much of a story was recalled, but to conduct a careful analysis of the *accuracy* of recalled information. That is, when speakers retell the gist and details from a fictional story they have heard, are they accurate? Overall, results illustrated that while some age differences exist in accurate recall of story information, these are limited to specific types (*i.e.*, categories) of information. Sometimes, but not always, these age differences are explained by differences in younger and older adults’ education and episodic memory ability. Interestingly, the influences of both age, and education and episodic memory ability, were trumped by gender, which was more informative as to whether an individual would accurately remember information or not.

#### 3.3.1. Age Effects

Both older and younger adults were equally accurate in the *overall* recall of gist and detail information from the stories they heard. Importantly, individuals of all ages recalled story information with a fairly high degree of accuracy, certainly sufficient for storytelling in everyday life (which the socially-relevant stories from this study were intended to reflect), where complete accuracy is not especially critical. When gist and detail were broken down into categories, however, age differences emerged. Older adults more accurately recalled *when* the event happened relative to younger adults, whereas younger people more accurately recalled *why* something happened in the story they heard. However, younger and older adults were equally accurate in remembering *who* was involved in the story, *where* the event occurred, and *what* happened.

##### Age Differences in Gist Recall

Why might older adults have had more accurate memory for *when* an event occurred, while younger adults had more accurate memory for the gist of *why* it occurred? Research on storytelling has indicated that while remembering all of the information from a story might be ideal, only specific aspects of the story may be necessary to convey the basic message. Information related to *who*, *what*, and *where* is generally considered essential [30,85], and these showed no age differences. This implies that when older and younger adults remember stories, they are equally accurate in those basic features of the stories that might matter most for conveying the story gist. *When* and *why*, however, may be non-canonical features of story recall [30,85]. This has implications for interpreting the age differences in accuracy observed here.

First, past research has shown that older adults have greater difficulty than younger adults when it comes to providing the dates for non-autobiographical events [86]. This initially seems incompatible with our result that older adults more accurately remember *when* an event occurred. However, recalling *when* the event occurred involved the recall of general information about event timing (e.g., the event occurred in the evening) rather than specific dates (e.g., 4th July). Older adults’ enhanced semantic knowledge about the world and high level of accumulated personal experience [7] may allow them to more accurately integrate this general *when* information with the rest of the story (*i.e.*, romantic events often occur in the *evening*, vacations often take place in the *summer*). Having this knowledge may have served to enrich the encoding process for older adults about when the event occurred, making it easier to accurately recall despite their difficulty in remembering exact dates.

As with recalling *when*, recalling *why* an event occurred may be peripheral to a story. Accurately recalling *why* may be less important than more central gist components. Research on story recall has shown that older adults, however, are actually more likely than younger adults to focus on the integrative or deeper meaning of the text [10,14]. With these stories, older adults may have expanded on the *why* gist by producing information that infused meaning into why events have happened (*i.e.*, because they fit in to a life story of a character, or create coherence for a character in a story, *etc.*) without being constrained by whether it was literally true. This may result in a less accurate but potentially more entertaining story. Older adults have been shown to tell better stories [87,88,89]. Part of that may be due to not being constrained by the literal retelling of *why* something happened.

Alternatively, understanding *why* an event occurred is less concrete than *what*, *who*, *when*, or *where*, and may require cognitive skills such as episodic memory to integrate when recalling a story. Indeed, our results somewhat support the idea that differences in cognition can at least partly explain age differences in gist-level accuracy for when and why fictional events occur. As was expected, age was negatively related to episodic memory performance and level of education, and controlling for these variables removed age differences in the accuracy of *when* and *why* gist categories.

##### Age Differences in Detail Recall

Education and episodic memory, although unrelated to the amount of gist recalled from the stories, were related to more accurately remembered details overall; specifically, better episodic memory was related to more accurately recalling details overall. However, controlling for education and episodic memory did not eliminate age differences in the accuracy of *emotion/thought* details: Younger adults more accurately remembered *emotion/thought* details than older adults regardless of education and episodic memory. This finding is at odds with research demonstrating that older adults rate their autobiographical memories as more pleasant [90] and engage in more elaborative processing of positive than negative information ([91], as per Socioemotional Selectivity Theory, [92]). Since the stories in the present study related to positive events (*i.e.*, romantic evening and vacation), it might have been expected that older adults would engage in more elaborative rehearsal for *emotion/thought* information, resulting in greater accuracy.

Our finding, however, parallels research on recall of autobiographical stories showing that younger adults produce more emotion/thought details than older adults [7]. As in Levine *et al.*’s [7] research, our category included both emotion and thought details. Any potential memory benefits for emotional information may have been neutralized by older adults' reduced recall of details overall, and particularly details related to thoughts. For example, Khanjani and colleagues [93] found that older adults were less able to identify with the mental states of others (*i.e.*, *thoughts*) relative to younger adults and less capable of interpreting emotional cues in others. This may suggest a decrease in cognitive perspective-taking that has implications for the recall of detailed thought and emotional information in stories about other people.

There were no other age differences in detail accuracy (*i.e.*, for *time*, *place*, or *perceptual* details). However, inclusion of an additional measure of accuracy, number of details omitted, revealed additional age differences: Older adults omitted more details than younger adults when retelling the stories they had heard, but this was driven by specific categories of information, not across all categories. Specifically, they recalled fewer *perceptual* and *emotion*/*thought* details, and these effects were maintained even when controlling for covariates (*i.e.*, were not due to episodic memory or education). These results parallel results from our accuracy analyses, in which older adults were less accurate when producing *emotion*/*thought* details, but critically, also expand on previous results by identifying another age difference (*perceptual*) that was not detected by just examining the accuracy of details alone. These results are also in line with past literature: Analogous research on autobiographical memory recall [7] has shown that younger adults produce more details related to *perceptual* information and *emotion/thought* relative to older adults. Thus, in this way, it is unsurprising that older adults omitted more *emotion/thought* and *perceptual* details in the present study.

In sum, compared to younger adults, older adults only have difficulty accurately recalling *perceptual* and *emotion*/*thought* details: information related to the fictional characters’ inner world (*i.e.*, perception, thought, emotion). This was most consistent for story characters’ emotions/thoughts (as indicated by age differences in *both* lower accuracy and higher rates of omission).

#### 3.3.2. Gender Effects

In addition to age effects, there were gender differences in the overall accuracy of gist and detail recalled from the stories participants heard, with women being generally more accurate than men. Note that there were no age-gender interactions. That is, obtained effects hold equally for older and younger people of the same gender. Specifically, women more accurately remembered the gist of *when* an event occurred, even when controlling for their higher scores on episodic memory. Paralleling this result, they also had better memory for details related to *time* (*i.e.*, details surrounding timing of the event) and *perceptual* features (*i.e.*, details related to the sensory landscape described in the story). Men were also more likely to omit *perceptual* and *time* details than women. Whereas women’s more accurate memory for the *when* gist remained when controlling for education and episodic memory, their superior memory for *time* and *perceptual* details disappeared after education and episodic memory were accounted for. It is possible that higher cognitive status, specifically women’s higher episodic memory, assisted in the retrieval of highly specified, unique details related to *time* and *perceptual* information such that controlling for education and episodic memory eliminated the gender differences. However, the retrieval of information related to the gist of *when* is broader, less specified, and more general, meaning that high functioning episodic memory may be less critical for recalling this information, and gender differences may be driven (at least up to a point, in tandem with cognitive status) by some additional, psychosocial factor.

The findings that women were more accurate than men on multiple dimensions of story recall is consistent with past research [58] and of the view of women as socialized to be the “record-keepers” of relationships, and therefore expected to remember key pieces of social information (e.g., the details of a birthday party or a cherished anniversary). This gender effect was particularly found for recalling the gist of *when* an event occurred and details related to *time* and *perceptual* information. The stories participants recalled were true-to-life scenarios that all participants were likely to have experienced themselves and heard about others experiencing as part of their daily lives. Note, however, that the topics—a vacation and romantic evening—clearly focused on social events. Previous research has demonstrated that women have more detailed, vivid, and accurate memories of social events than men, and men tend to forget or exclude more information [94]. Men, when asked to recall memories about shared social events, were more likely to request help from their wives than vice versa [59]. As such, our results are in line with past research suggesting that socially-salient stories about others optimize accurate recall for women regardless of their age.

#### 3.3.3. Limitations

The findings of this study shed light on how individual characteristics, such as age, gender, and education and episodic memory, relate to the accuracy of recall for non-autobiographical stories. However, there are several other factors, such as the types of stories and contexts under which a story is being recalled, that could also affect accurate story recall across the lifespan. For example, knowing that a persons’ gender influences the degree to which they accurately recall stories, it would be interesting to examine whether these same differences emerge for recall of fictional stories that vary in topic orientation (e.g., traditionally male-oriented topics, like news or sports). Similarly, measuring an individual’s motivation to remember information may play a key role in accurate recall. As aforementioned, the fictional topics used in the present study may have been more salient to women and likewise, easier to encode and subsequently remember. Additionally, women may have been more motivated to remember information from these social stories because they may embrace the persona of the “record-keeper”. However, future research may find that male-oriented topics are easier for men to encode and remember. Under these circumstances, men may display higher accuracy of information, similar to the women in the present study. Thus, manipulation of topic orientation would shed more light on the role of how contextual variables, like interest or relevance of the topic as well as personal motivation, influence the importance of accuracy when recalling stories.

Similarly, the context under which the story is recalled may influence how important accuracy of the story is in that moment. If the purpose of the story is to entertain the listener or facilitate social bonding, slight embellishments or the inclusion of inaccurate supplemental information may serve to enhance the story quality. Research from Barber and Mather [23] support this idea. In this study, they found that when older adults were explicitly told to focus on accuracy when initially retelling stories, they later recalled information from the story more accurately than older adults who initially retold the story for the purpose of being entertaining. Younger adults’ accuracy did not differ as a function of whether they were initially told to focus on being entertaining or accurate. However, it still remains to be seen whether the story context may even further dictate *which* specific pieces of information (e.g., *who*, *what*, *where*, *when*, or *why*) a person must accurately recall that are critical to the story. For example, in the hypothetical case where a person is describing an injury to a doctor, it may be more crucial to explain *what*, *where*, *when*, and *why* an accident occurred (*fell*, *down the stairs*, *today*, *slipped*) rather than *who* all was involved (*my sister saw it happen*). On the other hand, in the hypothetical case of a witnessed crime, it might be less important to accurately recall *why* the event occurred (the individual might not even have knowledge of that information) and more important to accurately recall *who* was involved, *what* crime occurred, and *when*/*where* it occurred. In sum, future research would benefit from developing a greater understanding how person-environment fit influences the degree to which individuals value accuracy during storytelling.

An additional limitation of this study relates to the role of ethnic diversity in narrative recall. Undoubtedly, cultural influences alter the ways in which both fictional and autobiographical stories are told as well as the function that these stories serve [95]. For example, research has shown that Asian American parents often use personal storytelling to help children identify with their culture of origin as well as the new American culture [96]. It is possible that accuracy is not as vital for stories of these kind, which are used to impart values, morals, or deeper understanding, at least for older adults who are often the ones telling these types of stories [97]. Participants in this study were predominantly Caucasian and all were American. The cultural generalizability of these results should therefore be considered.

Additionally, the between-subjects design does not distinguish between age and cohort effects, such that age (and gender) differences may be at least partly reflective of differences in culture/socialization histories. For example, one possibility is that younger cohorts may have greater educational opportunities than older cohorts (see [98] for a review). Further, the younger adults in the present study were college students, for whom knowledge-acquisition was especially salient. It is possible that in general, accuracy is of greater importance for these younger adults, given their current academic pursuits and historical differences in educational opportunities. This cohort difference would also be consistent with the hypothesis proposed by Socioemotional Selectivity Theory, which states that younger adults are more likely to engage in activities that are focused on knowledge-acquisition than older adults, who tend to engage in activities that promote social bonding [92]. However, it remains an open research question as to why specific categories of gist and detail recall would be more susceptible to cohort differences than others. On an additional note, it does not seem likely that gender differences were influenced by age cohort (*i.e.*, the fact that older women were socialized differently than younger women, such that younger women are less likely to fill traditional economic and social gender roles) [99]. Because gender did not interact with age to influence accurate recall (gender effects were consistent across age groups), the results do not seem to suggest that cohort effects differentially influenced younger and older women and men.

A final potential limitation of our study is a methodological one. Some past research using list recall [100] has indicated that when tested by a younger adult experimenter, older (but not younger) adults exhibit a stress response, leading to lower memory performance. Thus, one possibility is that older adults’ poorer recall performance may have been partly driven by feelings of stress when being interviewed by a young experimenter. However, we do not believe this to be the case for several reasons. First, previous storytelling research [101] suggests that younger and older women, for example, do not differentially elaborate when engaging with a young female experimenter, perhaps due the social nature of storytelling compared to list recall. This research has also illustrated that older adults are better able than younger adults to adjust their storytelling (e.g., their speaking style) to fit the developmental level of their audience [101], suggesting that older adults may be less sensitive to the mere presence of an experimenter who is of a particular age (*i.e.*, the listener) during storytelling. Second, as aforementioned, the interviewer acted as an interested listener who responded with engaging facial expressions and small agreements, thus reducing any potential stress that older adults may have experienced due to the interviewer’s demeanor. Finally, the lack of age differences in overall gist and detail recall does not support the claim that testing environment differentially influenced older adults’ performance; that is, if stress was a driving factor for age differences, it is unclear why only specific categories of gist and detail would be affected and not all categories similarly.

## 4. Conclusions

The present research is in line with lifespan psychology principles suggesting that aging brings multidirectional and multidimensional change [102,103]. In accurately recalling the gist and detail of socially relevant stories they had heard, older adults sometimes performed better, sometimes worse, and sometimes the same as younger persons. Further, cognitive decline is often thought to guide older adults’ performance on everyday tasks such as storytelling. While episodic memory ability and education did account for some age findings, that was not exclusively true. Examining communication styles and motivations for storytelling may be another way to account for age differences [8,87,101]. This approach also guides us to look at the person more holistically, not only in terms of their age. Other characteristics may also be important to story recall and sharing. The present study highlighted the relative importance of gender and age: Gender differences were more robust than age differences. Stories that we hear or read, and then tell and retell to others may not always be completely accurate but they are an important part of our social communication in daily life. Understanding how older and younger men and women recall different types of stories across different life contexts is an area for future research.
